# Case Report: Recurrent hypokalemic periodic paralysis associated with distal renal tubular acidosis (type 1) and hypothyroidism secondary to Hashimoto's thyroiditis

**DOI:** 10.12688/f1000research.15662.3

**Published:** 2019-01-15

**Authors:** E. Dante Meregildo-Rodríguez, Virgilio E. Failoc-Rojas

**Affiliations:** 1 Servicio de Medicina Interna y emergencia, Hospital Regional Lambayeque, Lambayeque, Peru; 2 Unidad de Investigación para la Generación y Síntesis de Evidencias en Salud, Universidad San Ignacio de Loyola, Lima, Peru

**Keywords:** hypokalemic periodic paralysis; distal renal tubular acidosis (type 1); hypothyroidism; Hashimoto Disease

## Abstract

**Background:** Hypokalemic periodic paralysis (HypoKPP) is characterized by transient episodes of flaccid muscle weakness. We describe the case of a teenaged boy with HypoKPP and hyperthyroidism due to Hashimoto's thyroiditis with initial manifestation of renal tubular acidosis. This combination is rare and little described previously in men.

**Case presentation**: A 17-year-old boy was admitted after three days of muscular weakness and paresthesia in the lower limbs with an ascending evolution, leading to prostration. Decreased strength was found in the lower limbs without a defined sensory level, reduced patellar and ankle reflexes. Positive antithyroid antibodies were found. He received hydration treatment, IV potassium and levothyroxine, with which there was a clinical improvement. Other examinations led to the diagnosis of type 1 renal tubular acidosis.

**Conclusion**: HypoKPP is a rare disorder characterized by acute episodes of muscle weakness. Type 1 renal tubular acidosis can occur as a consequence of thyroiditis, which is explained by the loss of potassium. This combination is unusually rare, and has not been described before in men. The etiopathogenesis of the disease as well as a dynamic explanation of what happened with the patient are discussed in this report.

## Abbreviations

HypoKPP: hypokalemic periodic paralysis; RTA: renal tubular acidosis; DRTA: distal renal tubular acidosis

## Introduction

Hypokalemic periodic paralysis (HypoKPP) is an uncommon neuromuscular disorder characterized by transient episodes of flaccid muscle weakness, and exceptionally, respiratory failure and death ^1, 2^. In addition, HypoKPP is the most common type of periodic paralysis, and may be primary (familial or idiopathic) or secondary (acquired) ^2– 4^. Primary HypoKPP occurs when the channelopathies produce potassium intracellular translocation. Secondary HypoKPP is caused by the loss of potassium from kidneys, gastrointestinal tract or skin ^1, 5– 9^. HypoKPP cases related to thyroid disorders, more frequently thyrotoxicosis, and several autoimmune diseases have been previously reported ^2, 3, 5^.

Renal tubular acidosis (RTA) is defined as the failure of kidneys to acidify the urine when the glomerular filtration rate is normal or almost normal ^3, 9– 11^.

We report the case of a teenaged boy with HypoKPP and hypothyroidism due to Hashimoto's thyroiditis with initial manifestation of RTA. This combination is rare and previously has been reported in women ^3, 9– 12^, and few cases in males ^13^.

## Case report

A 17-year-old boy, from the region of Cajamarca, high Andean area of Peru, without any relevant medical personal or familial history, was admitted to the Lambayeque Regional Hospital in April, 2017. For three days the patient had muscle weakness and paresthesia in the lower limbs with an ascending evolution and proximal predominance that made his condition worse, leading to prostration and arrival by emergency route. The patient arrived at the hospital awake, hemodynamically stable, with 24 rpm tachypnea. Thyroid gland was not palpable. A neurological physical examination showed weakness in the lower limbs without a defined sensory level, and reduced patellar and ankle reflexes. There was no evidence of bulbar muscle, respiratory and sphincter involvement.

Regarding serum electrolytes upon admission, they showed hypokalemia (1.44 mmol/L [normal values NV: 3.5–4.5 mmol/L]) without sodium, chloride or calcium alterations. Regarding the ancillary examinations upon admission: hematology tests were within the normal range; normal biochemistry values; elevated thyroid stimulating hormone (TSH) of 5.5 mU/ml ([NV]: 0.27–4.2 mU/ml); low free T4 of 0.78 ng/dl (NV: 0.9–1.7 ng/dl).

The patient was evaluated by the Department of Nephrology, Endocrinology and Neurology and diagnosed with hypothyroidism and hypokalemia. He received replacement treatment with normal saline solution, IV potassium and levothyroxine (T4) 25ug/day. On the fourth day of the treatment, he showed normal potassium values (3.7 mmol/L). After the patient’s clinical condition improved, one week after his admission to the hospital, he was discharged with diagnoses of hypothyroidism (etiology to be determined) and hypokalemia resolved.

Around five weeks after the patient was discharged, he was examined in the endocrinology office and did not show any symptoms. He was indicated to continue with T4 at 25ug/d. Glucose, urea, creatinine, prolactin, morning serum cortisol, testosterone, follicular stimulating hormone, TSH, and free T4 were determined in serum; all of them within normal range. Antithyroid antibodies were positive: anti-thyroglobulin 445.5 UI/ml, anti-TPO 48.20 UI/ml. The following elements were analyzed in 24 hour urine sample: sodium 255.66 mEq., chloride 55.1 mEq., and potassium 89 mEq. Urine test: leukocytes 1–3/field, red blood cells 1–3/field, density 100.5, pH 8, glucose, blood, proteins and leukocyte esterase, all of them negative. Kidney echography: Kidneys maintained their size with multiple images compatible with nephrocalcinosis, bilateral and vesical renal lithiasis. Contrast-enhanced CT of sella turcica was normal. Electrocardiogram: bradycardia (heart rate 52 bpm), signs compatible with hypokalemia (ST-segment descent, prominent U waves and pseudo-prolongation of the QT interval). No low voltage complexes were observed.

**Figure 1.**
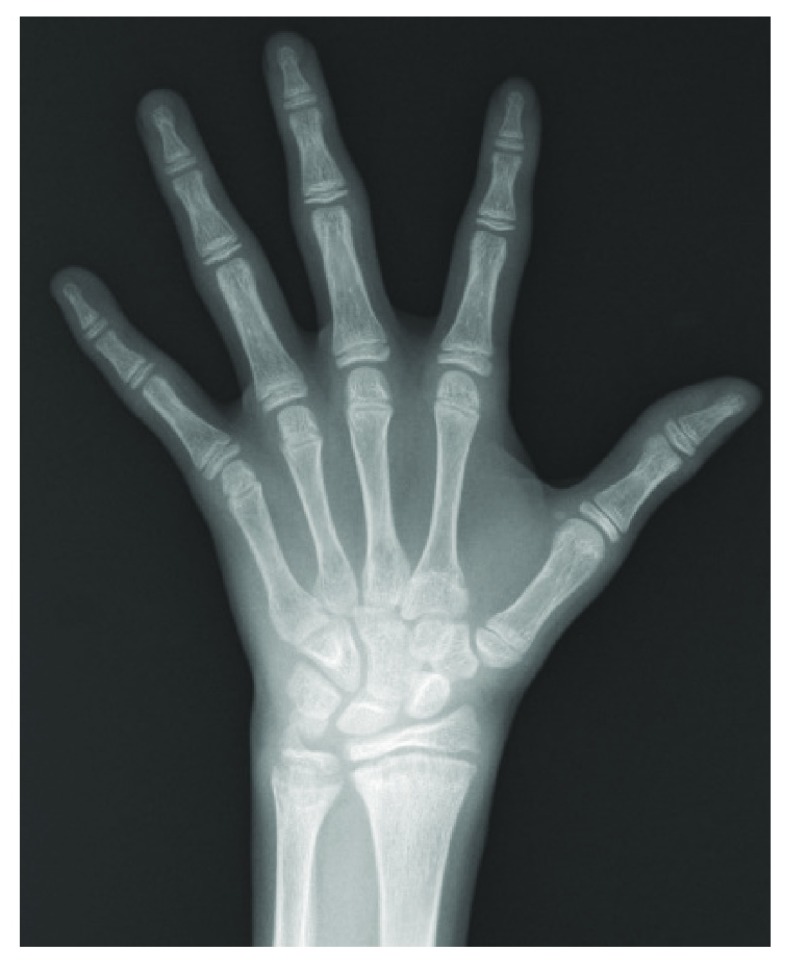
X-ray of the left hand of the patient with hypokalemic periodic paralysis. It showed a bone at the age of 13 years and 6 months.

A left-sided X-ray ( Figure 1) shows the bone age corresponding to 13 years and 6 months according to the Greulich and Pyle method ^14^. From this, it can be concluded that the diagnosis is secondary hypothyroidism to thyroiditis and distal RTA.

Three months after the first episode, over a three week period, the patient stopped the intake of levothyroxine. Around one week later, a new episode of progressive muscle weakness occurred again, which was similar to the previous one. Two days later, the patient was evaluated at a private consultation. The administration of T4 at 50 ug/day was recommend. The symptoms continued progressing, and 5 days later, the patient was admitted again to our hospital by emergency presenting with flaccid quadriparesis with predominance in the lower limbs. 

Laboratory tests on second admission: normal hematology values; normal biochemistry values. Urine tests: leukocytes 10–12/field, red blood cells 1–3/field, density 100.5, pH 8, glucose, blood, proteins, and leukocyte esterase, all of them negative. No provocation tests or genetic studies were performed in search of channelopathies. A urine culture and a thorax x-ray were conducted, and both of them showed a normal outcome. Blood gases and serial electrolytes tests shown in Table 1 were also performed. Periodic hypokalemic paralysis was diagnosed in addition to the previous diagnoses.

**Table 1.** Blood gases and electrolytes in the second admission of the patient at two time points.

	07-2017	07-2017 (72 hours later)
BGA (FiO2 0,21)		
pH (7.35-7.45)	7.22	7.35
pO _2_ (75-100 mmHg)	103.8	106.4
PCO _2_ (35-45 mmHg)	22.8	24.3
HCO _3_ (22-28 mmol/L)	13.9	21.0
Na ^+^ (135-145 mmol/L)	141.3	144.5
K ^+^ (3.5-5.0 mmol/L)	1.98	4.07
Cl ^-^ (98-106 mmol/L)	118.1	115.0
Ca ^2+^ (1.10-1.35 mmol/L)	1.09	1.25
Mg ^2+^ (0.65-1.05 mmol/L)	0.54	0.51
EB (-2 to +2 mmol/L)	-10.9	-4.7
GAP (8-12 mmol/L)	9.33	8.5

**BGA: Blood Gas Analysis**

He received treatment with IV potassium, IV sodium bicarbonate and T4 25ug/day. The patient improved clinically and was discharged at day five, with T4 25 ug/day. We could not analyze trans-tubular potassium gradient because this test was not available in our hospital. Follow up is every three months with nephrology and internal medicine departments. After continued treatment with T4 was maintained by the patient, no additional oral bicarbonate administration was necessary.

## Discussion and conclusions

Thyrotoxicosis is the most common secondary cause of HypoKPP; while hypothyroidism is an extremely rare cause of HypoKPP ^6, 8^.

RTA is a rare cause of HypoKPP ^6, 9– 12^. Table 5 in the paper by Rodríguez Soriano (2002) ^15^ summarizes the different characteristics of types of RTA, leading us to conclude that our patient had distal RTA (DRTA). DRTA is the most common form of RTA. In adults, the most common causes of DRTA are autoimmune disorders such as hyperthyroidism, hypothyroidism, Hashimoto’s thyroiditis, rheumatoid arthritis, Sjögren syndrome, systemic lupus erythematosus, and type 1 diabetes mellitus ^3– 5^. However, autoimmune diseases rarely cause DRTA with severe hypokalemia ^2– 4^, but this was observed in the case we report here.

The excretion of H ^+^ is carried out by the intercalated alpha cells (type A) of the distal nephron (distal convoluted tubule and cortical collecting tubule) by means of H ^+^-ATPase luminal pumps, and to a lesser extent, H ^+^-K ^+^-ATPase pumps. The secretion of K ^+^, is carried out fundamentally, by the main cells in the collecting tubules ^10, 11^. In hypothyroidism, the number and functioning of these pumps are reduced, which causes a reduction of the excretion of H ^+^, exacerbating the acidosis produced by RTA. The treatment with thyroid hormone increases the activity of these cellular pumps ^4, 12^. Since Hashimoto’s thyroiditis is the most common cause of hypothyroidism, DRTA could be an underdiagnosed condition associated ^12^. DRTA associated with non-autoimmune hyperthyroidism has been also described ^3^.

It is most common that the hereditary forms of DRTA produce bone demineralization, which can cause osteoporosis and osteomalacia in adults, and rickets and growth delay in children ^3, 4, 10, 12^. A bone age retardation of >2 years, in the absence of endocrine deficiency, suggests a constitutional growth delay; while a bone age retardation >3 years is considered pathological ^11, 16^. Our patient showed a bone age >3 years.

DRTA is associated with hypercalciuria, hypocitraturia, nephrolithiasis, nephrocalcinosis, chronic interstitial nephritis, and progressive renal failure ^3– 5, 10, 12^. Severe complications of chronic acidosis like myocardial failure, lethargy and coma are rare ^9^. Although HypoKPP associated with P(proximal)RTA have been previously described, the HypoKPP associated with DRTA is more common, more abrupt and more severe ^7^.

In HypoKPP, whether by potassium movement into cells or by potassium loss, the resulting hypokalemia reduces the resting membrane potential and blocks the action potential ^1, 2^. The paralytic attacks occur suddenly with localized or generalized weakness, and can last from one hour to days ^1, 6^. Muscle weakness is predominantly proximal and more in the legs than arms, and hyporeflexia is typical. Sensitivity and consciousness are preserved and it is uncommon that extra-ocular, facial, bulbar and sphincter muscles are involved ^1, 2, 4^.

ECGs can show signs of hypokalemia, including ST depression, decrease of the amplitude of the T-wave and an increase of the amplitude of the U-waves; but arrhythmias such as atrial fibrillation, supraventricular paroxysmal tachycardia, or ventricular fibrillation are not common ^1, 6^. Our patient showed pseudo-QT prolongation due to the presence of U-wave in the QT segment. However, when QT is measured in aVL, where the U-wave is less prominent, the actual QTc value is obtained and it was normal.

In the present case, we didn’t find any other obvious cause of hypokalemia, apart from the DRTA. The possibility of hypokalemic tubulopathies losing salt (Gitelman syndrome and Bartter syndrome) are less probable since they typically present with metabolic alkalosis, hyponatremia and elevated potassium levels; and also because there was no known familial history of any kidney disorder. Other rare causes of periodic paralysis that occur with hypokalemia, such as thyrotoxic periodic paralysis and Andersen's syndrome, are unlikely given the case presentation. The first of these disorders present with thyrotoxicosis and the second presents with dysmorphic features, ventricular arrhythmias, and a long QT interval with normal, high, or low serum potassium ^1^.

In our patient, two secondary HypoKPP causes that were extremely rare were found, the secondary hypothyroidism to Hashimoto’s thyroiditis and distal RTA. HypoKPP and DRTA diagnosis was established for that patient with a clinical condition associated with severe hypokalemia. The DRTA diagnosis is fully supported by the presence of hyperchloremic metabolic acidosis, severe hypokalemia, alkaline urine, positive urine anion gap, nephrolithiasis, nephrocalcinosis and polyuria, with normal renal function. In addition, according to the anthropometry of the patient, he failed to thrive and had a bone age retardation of 3.5 years, both conditions typically associated with DRTA. The diagnosis of autoimmune hypothyroidism is evident due to the results of the thyroid profile.

DRTA requires the administration of alkaline salts to correct the acidosis. Sodium bicarbonate should be administered again (1–2 mEq/kg/d) to help to satisfy the alkali requirements and compensate the bicarbonate loss ^4, 5^. In addition, oral and IV potassium must be administered again. Our patient responded well to the oral and IV potassium, IV sodium bicarbonate and thyroid hormone replacement treatment. The different differential diagnoses in patients with muscle weakness episodes should be taken into account. Despite not having provocation tests or genetic tests, the clinic and laboratory led to the success of the treatment

## Consent

Written informed consent was obtained from the patient for publication of this case report and any accompanying images.

## Data availability

All data underlying the results are available as part of the article and no additional source data are required.
