# Supplementary material for: Case Report: Recurrent hypokalemic periodic paralysis associated with distal renal tubular acidosis (type 1) and hypothyroidism secondary to Hashimoto's thyroiditis
Source: F1000Res. 2019 Jan 15;7:1154. Originally published 2018 Jul 30. [Version 3] doi: 10.12688/f1000research.15662.3 (PMC6325611; doi:10.12688/f1000research.15662.3)
Supplement: Peer review history

# Peer review: Referee response for version 2

**DOI:** 10.5256/f1000research.19283.r42522

**Published:** 2019-01-07

**Authors:** Stalin Viswanathan

**Affiliations:** 1 Department of General Medicine, JIPMER, Puducherry, Puducherry, India

The issues have been addressed. A minor two points:

- In the paragraph preceding the hand radiograph, it is mentioned as "Hashimoto's hyperthyroidism" instead of hypothyroidism.
- On many occasions, DRTA and DTAR have been used interchangeably. Uniform abbreviation should be adhered to.

I have read this submission. I believe that I have an appropriate level of expertise to confirm that it is of an acceptable scientific standard.

# Response

**Published:** 2019-01-11

**Authors:** Virgilio E Failoc-Rojas

**Affiliations:** Unidad de Investigación para la Generación y Síntesis de Evidencias en Salud, Universidad San Ignacio de Loyola, Lima, Perú, Peru

Thank you very much for your suggestions, they have been accepted and modified accordingly, the term DRTA is standardized.

# Peer review: Referee response for version 1

**DOI:** 10.5256/f1000research.17089.r41009

**Published:** 2018-11-26

**Authors:** Stalin Viswanathan

**Affiliations:** 1 Department of General Medicine, JIPMER, Puducherry, Puducherry, India

In the introduction, 3 ^rd^ paragraph: “… HypoKPP and hyperthyroidism due to Hashimoto…" is written instead of “hypothyroidism”.

 In the same paragraph: “This combination is rare and previously has only been reported in women”. Such a combination has been reported at least once previously in 2017 ^1^. Since I am one coauthor of this paper, I am not sure whether I can insist that this should be quoted.

 In the Case report section 2 ^nd^ paragraph:

- It would have been preferable to mention the hypokalemia first and then mention secondary investigations such as thyroid profile and magnesium/calcium.
- The TSH of 5.5 is normal for many labs. If it was slightly higher than your lab limits, it would be preferable to label it subclinical hypothyroidism, since TSH is <10.
- Family history of thyroid disease or other autoimmune diseases can be mentioned.
- Also, did the patient have a goitre?
- Did the ECG also show low voltage complexes or bradycardia suggestive of hypothyroidism?
- It is not mentioned whether he was treated with bicarbonate supplements on an outpatient basis when a diagnosis of RTA was made.
- Was TTKG considered if the facilities for urine osmolality were available?

In Table 1:

- Magnesium appears low in the table, but in page4/7 last paragraph, it is mentioned that the patient did not have hypomagnesemia and hence Gitelman was not considered.
- It is also mentioned that Gitelman will have elevated K levels which is untrue. Since the patient had high urine K loss and hypomagnesemia, Gitleman was considered except that ABG showed metabolic acidosis.

I have read this submission. I believe that I have an appropriate level of expertise to confirm that it is of an acceptable scientific standard, however I have significant reservations, as outlined above.

## References

[1] KadeejaNSenthilnathanNViswanathanSAghoramR: Sporadic hypothyroidism-related hypokalemic paralysis: Diagnosis in a resource-poor setting. *J Family Med Prim Care*.6(4) : 10.4103/jfmpc.jfmpc_215_17 862-864 10.4103/jfmpc.jfmpc_215_17 29564279PMC5848414

# Response

**Published:** 2018-12-16

**Authors:** Virgilio E Failoc-Rojas

**Affiliations:** Unidad de Investigación para la Generación y Síntesis de Evidencias en Salud, Universidad San Ignacio de Loyola, Lima, Perú, Peru

Dear Dr Stalin.

The editing corrections are corrected in the new version that will be sent.

In addition, the article that suggests will be cited.

There was no family or personal background. This additional information is added to the Case Report.

The patient did not have a goiter. This additional information is added to the Case Report.

The EKC showed bradycardia, but not low-voltage complexes. This additional information is added in the Case Report.

During follow-up after discharge, the patient maintained treatment with T4 and it was not necessary to administer oral bicarbonate.

It was considered, but it was not possible to analyze TTKG (transtubular potassium gradient) since this test is not available in our hospital. This additional information is added to the Case Report.

the result of the serum magnesium was 0.54 and 0.51 mmol / L (our laboratory considers a reference range of 0.65-1.05 mmol / L). Therefore, the patient did present hypomagnesemia.

The reviewer is right in stating that Gitelman syndrome cannot be ruled out, although it is true that the patient did not present with metabolic alkalosis, hyponatremia, and hyperkalemia if he presented hypomagnesemia and it is possible that due to the high losses of potassium in urine The patient will not have hyperkalemia. Therefore, the statement is corrected and reformulated: *In the presents case, we didn’t find any other obvious cause of hypokalemia, apart from the DRTA. The possibility of hypokalemic tubulopathies losing salt (Gitelman syndrome and Bartter syndrome) are less probable since they typically present with metabolic alkalosis, hyponatremia, and elevated potassium levels; and also because there was no known familial history of any kidney disorder”.*

# Peer review: Referee response for version 1

**DOI:** 10.5256/f1000research.17089.r37480

**Published:** 2018-10-31

**Authors:** Cristian Díaz-Vélez

**Affiliations:** 1 Centro de Investigación en Epidemiología Clínica y Medicina Basada en Evidencias, Instituto de Investigación, Facultad de Medicina Humana, Universidad de San Martín de Porres, Chiclayo, Peru; 2 Oficina de Inteligencia Sanitaria, Hospital Nacional Almanzor Aguinaga Asenjo EsSalud, Chiclayo, Peru

1. The case report is well written, light and fast reading.

 2. The values of potassium are displayed in moles and the normal value in another units, use the same units

 3. Specify the importance of bone age for the Case Report

 4. Try to explain the presence mostly in women

 5. Develop a diagram "decision making" with the presumptions diagnostic

I have read this submission. I believe that I have an appropriate level of expertise to confirm that it is of an acceptable scientific standard.

# Response

**Published:** 2018-12-16

**Authors:** Virgilio E Failoc-Rojas

**Affiliations:** Unidad de Investigación para la Generación y Síntesis de Evidencias en Salud, Universidad San Ignacio de Loyola, Lima, Perú, Peru

Dear Dr Cristian. 

Answering your questions.

1) For a new version, the reference values will be added and expressed in mmol/ L

2) The delays in bone maturation, evaluable by bone age and the failure of bone growth are typical alterations of distal renal tubular acidosis. This is explained in the penultimate paragraph.

3) Most autoimmune disorders, including are more common in women.
